# Supplementary material for: Microglial NF-κB drives tau spreading and toxicity in a mouse model of tauopathy
Source: Nat Commun. 2022 Apr 12;13:1969. doi: 10.1038/s41467-022-29552-6 (PMC9005658; doi:10.1038/s41467-022-29552-6)
Supplement: Supplementary file 1 — Supplementary Information [file 41467_2022_29552_MOESM1_ESM.pdf]

Supplementary Information

Microglial NF-κB drives tau spreading and toxicity in a mouse model of tauopathy

Chao Wang[1], Li Fan[2], Rabia R. Khawaja[3], Bangyan Liu[2], Lihong Zhan[1], Lay Kodama[2], Marcus Chin[1], Yaqiao Li[1], David Le[1], Yungui Zhou[1], Carlo Condello[4,6], Lea Grinberg[5,6], William W. Seeley[5,6], Bruce Miller[5,6], Sue-Ann Mok[7], Jason E. Gestwicki[4,8], Ana Maria Cuervo[3], Wenjie Luo[2*], Li Gan[1,2,9*]

Supplementary Figures 1–10

Supplementary Data 1–18

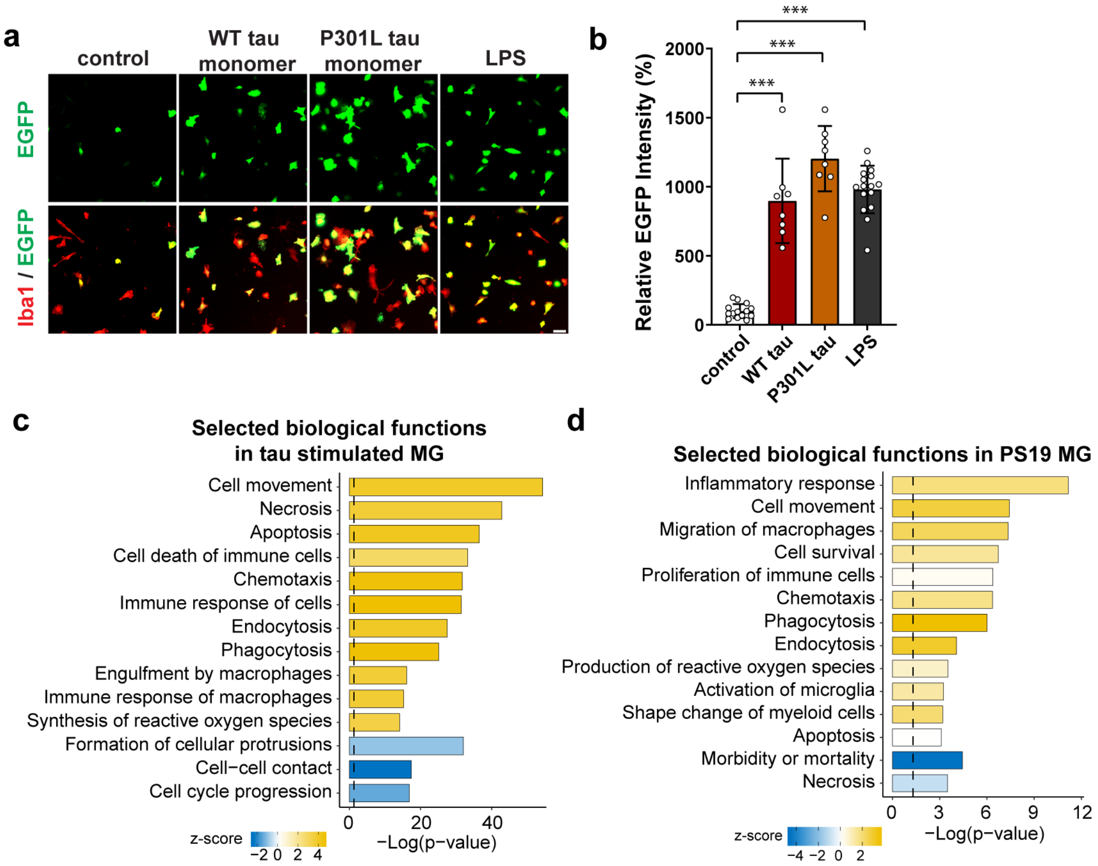

Supplementary Fig. 1(related to Fig. 1) Tau activates NF-κB pathway and modulates biological functions in microglia

(a,b) Primary microglia infected with NF-κB reporter (Lenti-κB-dEGFP) virus were incubated with wildtype (100nM), P301L (100nM) tau monomers and LPS (50ng/ml) for 24h. (**a**) Representative fluorescence high content images of EGFP (green) and Iba1 (red) Scale bar, 50μm; (**b**) Quantification of EGFP intensity. Values are mean ± SD, relative to vehicle control. Total N=14(control), 8(WT tau),8(P301L tau) and 16(LPS) wells from two independent experiments. P-value was calculated using multilevel mixed-effect model with experiment as hierarchical level, ***p<0.001. Source data are provided as a Source data file.

(c,d) Selected IPA biological functions identified for DEGs in tau stimulated (**c**) or PS19 (**d**) microglia. P-values were calculated using right-tailed Fisher's exact test with threshold of significant enrichment as p-value≤0.05 (indicated by a dotted line of -log (p-value) = 1.3).

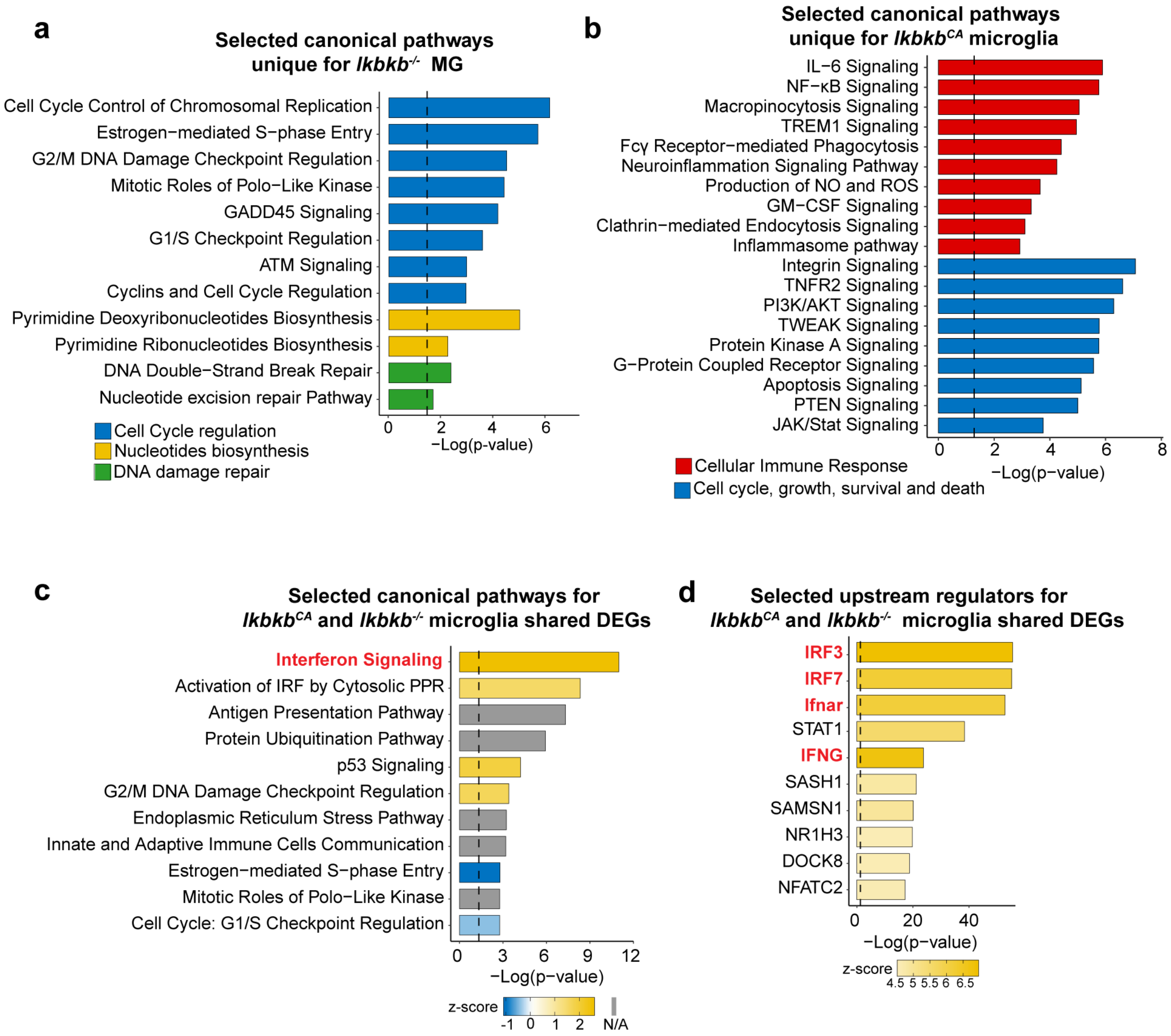

Supplementary Fig. 2 (related to Fig. 2) IPA analysis of unique and shared DEGs of *Ikbkb*$^{-/-}$ and *Ikbkb*^{CA} microglia

(a,b) Selected IPA canonical pathways identified for unique DEGs of *Ikbkb*$^{-/-}$ microglia (**a**) and *Ikbkb*^{CA} microglia (**b**). Canonical pathways are grouped by indicated categories.

(c,d) Selected IPA canonical pathways (**c**) and upstream regulators (**d**) identified for shared DEGs of *Ikbkb*$^{-/-}$ and *Ikbkb*^{CA} microglia. P-values were calculated using right-tailed Fisher's exact test with threshold of significant enrichment as p-value$\leqslant$0.05 (indicated by a dotted line of -log (p-value) = 1.3).

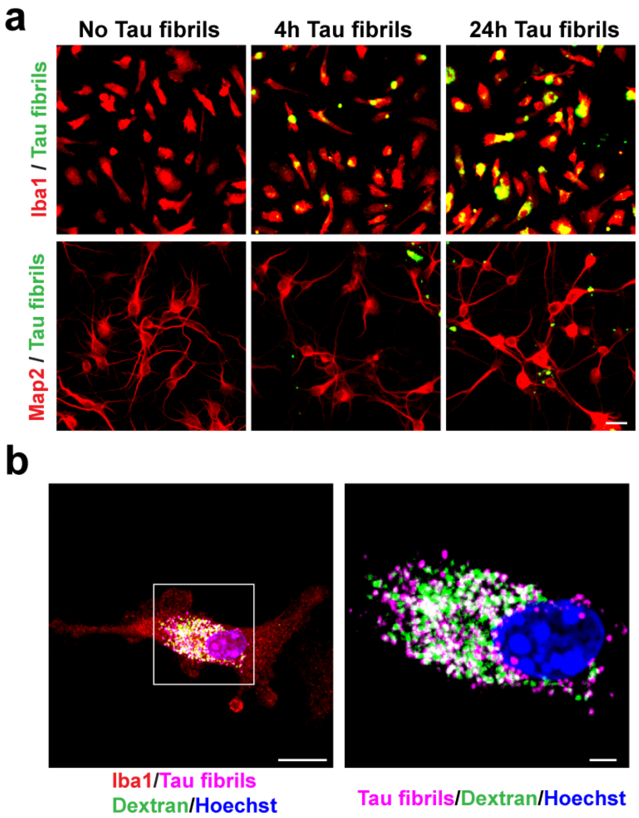

Supplementary Fig. 3 (related to Fig. 3) Microglia take up tau fibrils
(a) Primary microglia (Iba1+) and neurons (Map2+) were incubated with fluorescent tau fibrils for 4 and 24 hours. A representative image set from two independent experiments show that microglia, but not neurons, take up tau fibrils in a time-dependent manner. Scale bar, 25 µm.
(b) A representative image from two independent experiments of immunocytochemical staining show that tau fibrils were co-localized with lysosomes in microglia. Lysosomes were traced by Dextran-FITC, followed by incubation of fluorescent tau fibrils for 3 hours. Nuclei were labeled by Hoechst. Scale bar, left 10 µm, right 2 µm.

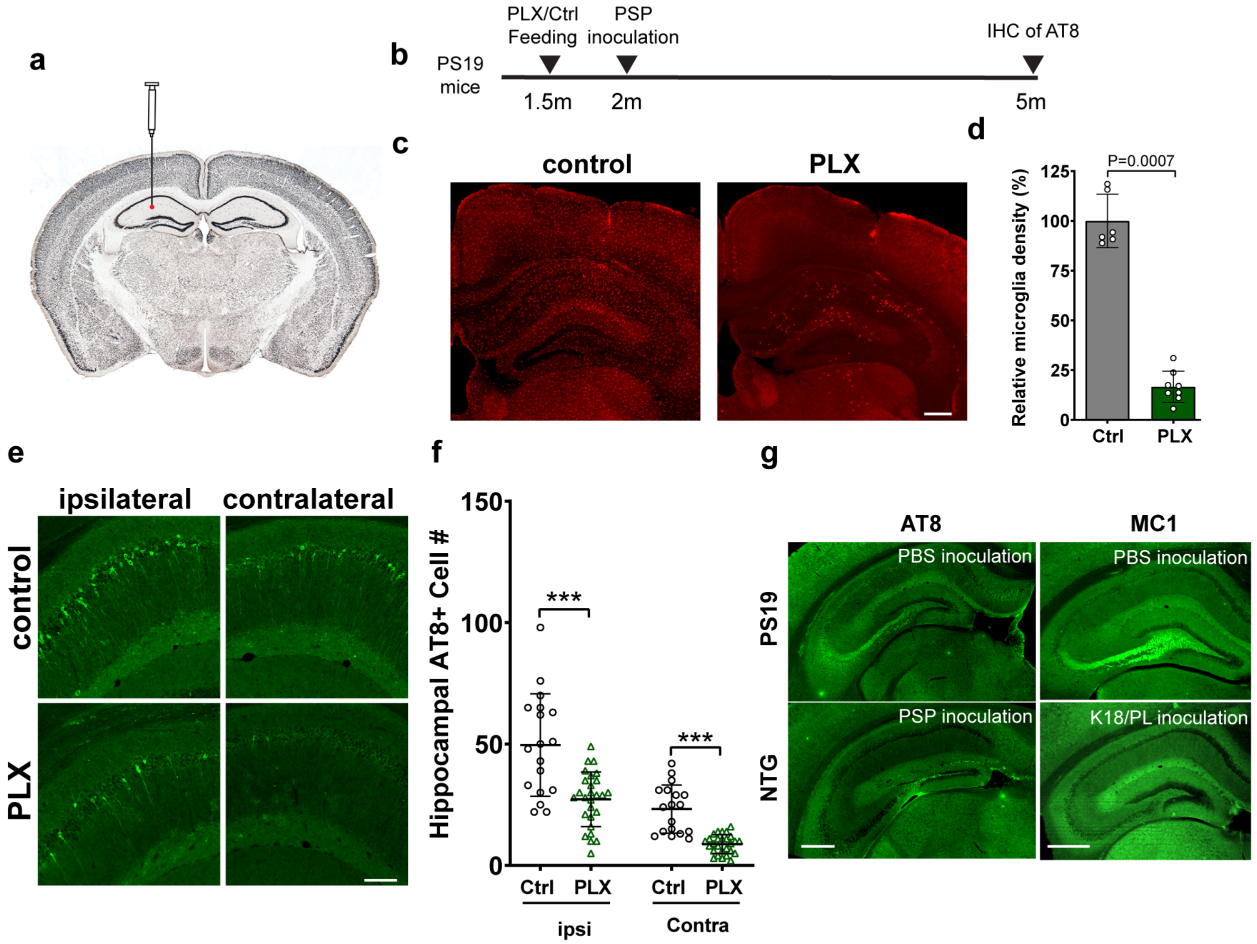

Supplementary Fig. 4 (related to Fig. 4) Depletion of microglia in PS19 mice halts tau seeding and spreading

(a) Schematic diagram illustrating tau seeds inoculation path. The red dot indicates injection site in hippocampus.

(b) Schematic diagram illustrating the experimental design and timeline of microglia depletion and PSP brain extract inoculation in PS19 mice. Pathological tau seeding and spreading was determined by immunohistochemical staining of AT8.

(c,d) Representative images (**c**) and quantification (**d**) of Iba1 positive microglia in control diet fed mice (n=6) and PLX diet fed mice (n=8). Values are mean ± SD, two-tailed Mann-Whitney test. Scale bar, 500µm

(e,f) Representative immunohistochemical staining of AT8 tau in ipsilateral and contralateral hippocampus CA1 region (**e**) and quantification of AT8+ neurons number (**f**) in control diet fed mice (n=6) and PLX diet fed mice (n=9), 3 sections per mice. Values are mean ± SD, P-values were calculated using multilevel mixed-effect model with mouse as hierarchical level. *** $p<0.001$. Scale bar, 200µm

(g) Left, representative immunohistochemical images of AT8 from four PS19 mice inoculated with PBS and from four non-transgenic mice inoculated with PSP brain extract; Right, representative immunohistochemical images of MC1 from four PS19 mice inoculated with PBS and from three non-transgenic mice inoculated with K18/PL tau fibrils. Scale bar, 500µm
Source data are provided as a Source data file

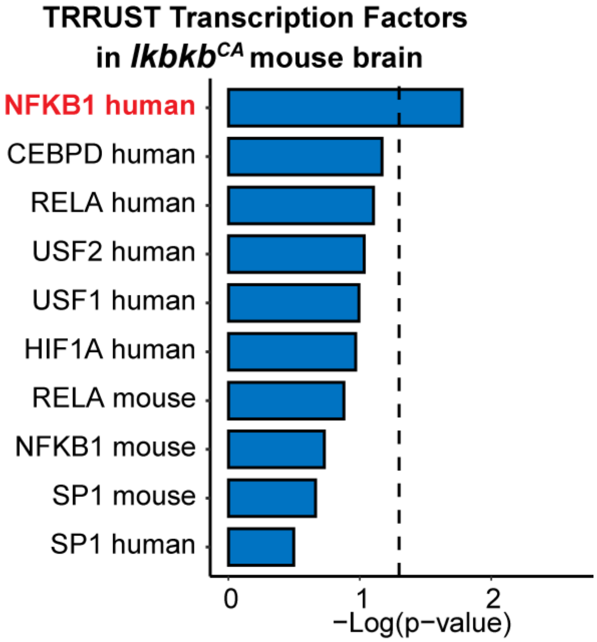

Supplementary Fig. 5 (related to Fig. 4) NF-κB is predicted as the top transcription factor in *Ikbkb*^{CA} mouse brain

Bulk RNA-seq were performed in cortical tissue of *Ikbkb*^{WT} and *Ikbkb*^{CA} mice (n=4). Upregulated DEGs (FDR<0.05, $Log_2FC>0$) were analyzed to predict potential transcription factors using TRRUST 2019 database. P-values were calculated using right-tailed Fisher's exact test with threshold of significant enrichment as p-value$\leqslant$0.05 (indicated by a dotted line of -log (p-value) = 1.3).

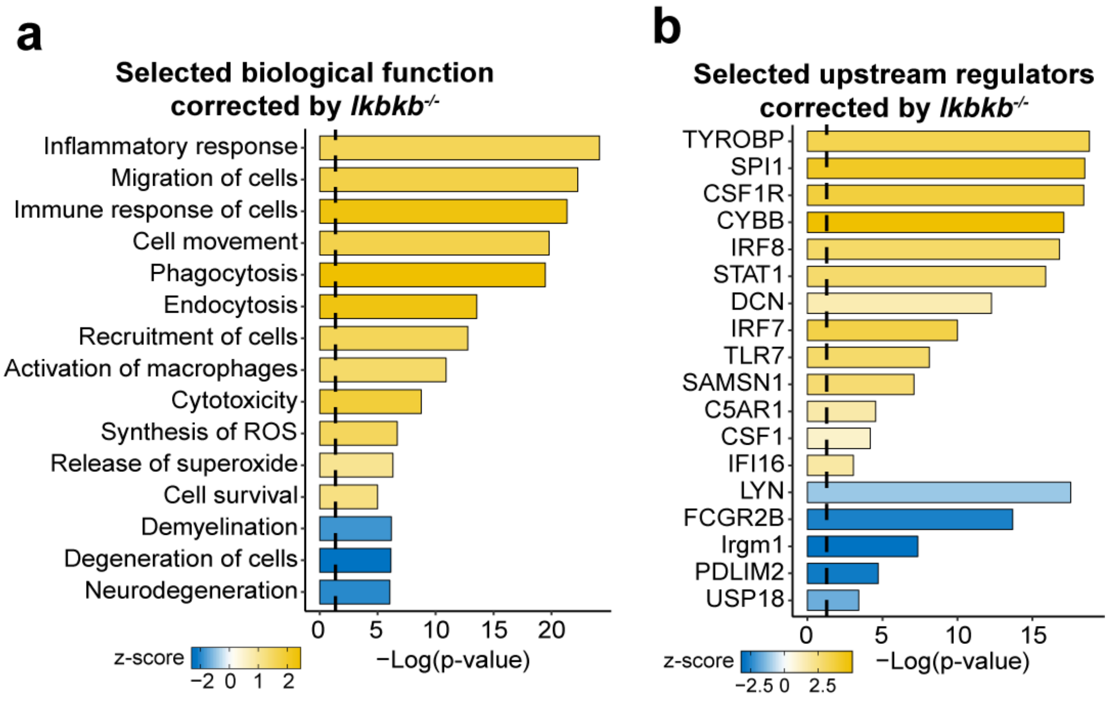

Supplementary Fig. 6 (related to Fig. 6) Inactivation of NF-κB rescued tau-mediated transcriptomic changes

Selected biological functions (**a**) and top predicted upstream regulators (**b**) identified for 286 DEGs corrected by microglial IKKβ deletion in PS19 mice that are shown in Fig. 6e. P-values were calculated using right-tailed Fisher's exact test with threshold of significant enrichment as p-value ≤ 0.05 (indicated by a dotted line of -log (p-value) = 1.3).

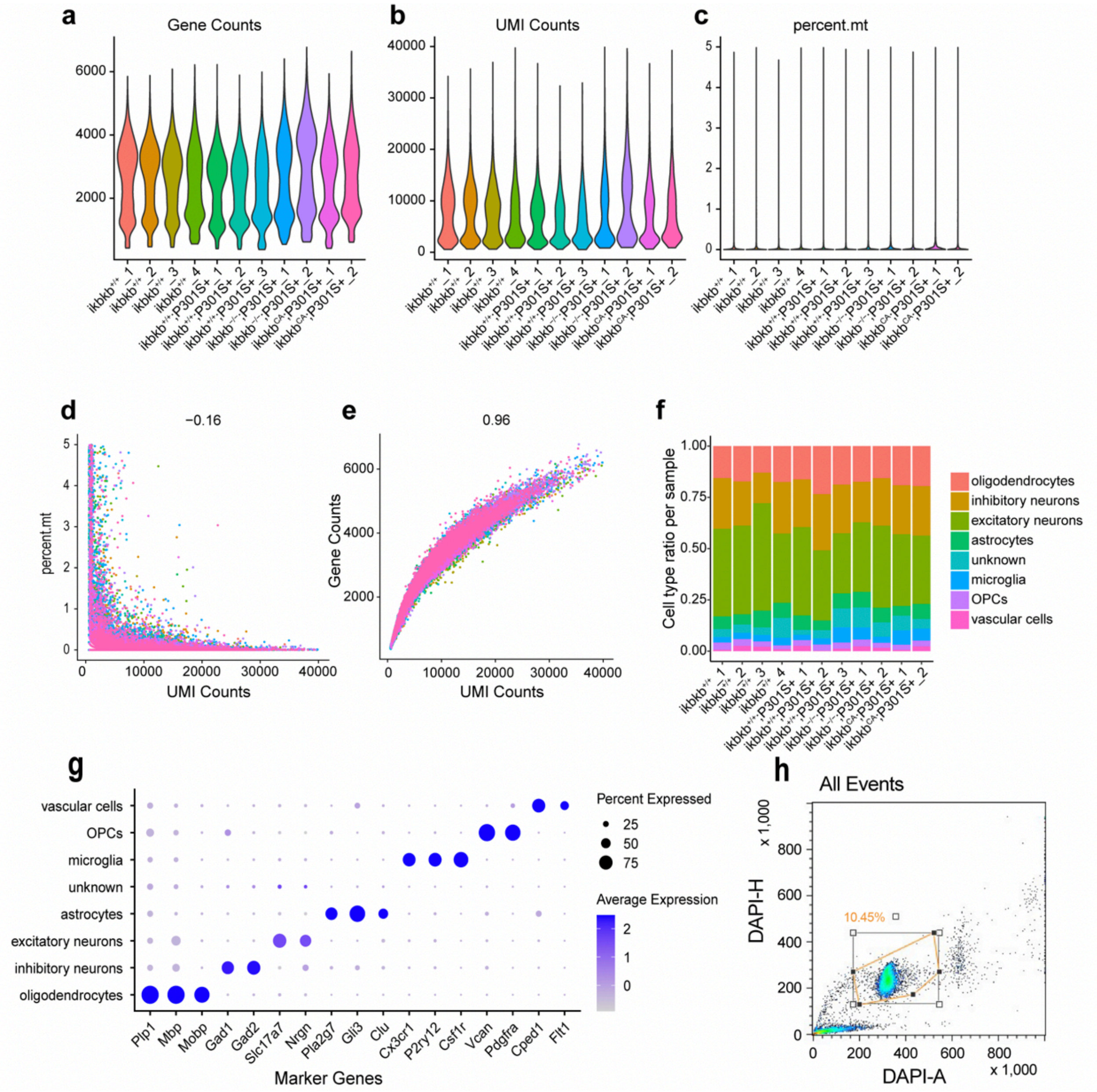

Supplementary Fig. 7 (related to Fig. 7) Quality control assessment of single nuclei RNA-Seq of cortical tissues

(a-c) Violin plots showing spread of total genes (**a**), total UMIs (**b**), and percent of mitochondrial genes (**c**) detected per nuclei for each individual sample.

(**d,e**) Correlation between UMI counts and percentage of mitochondrial genes per nuclei (**d**) and total genes detected (**e**) for all samples.

(f) Proportion of cell types for each individual sample.

(g) Percentage and average expression levels of maker genes for indicated cell types.

(h) FACS gating strategy for sorting singlet, DAPI-stained nuclei.

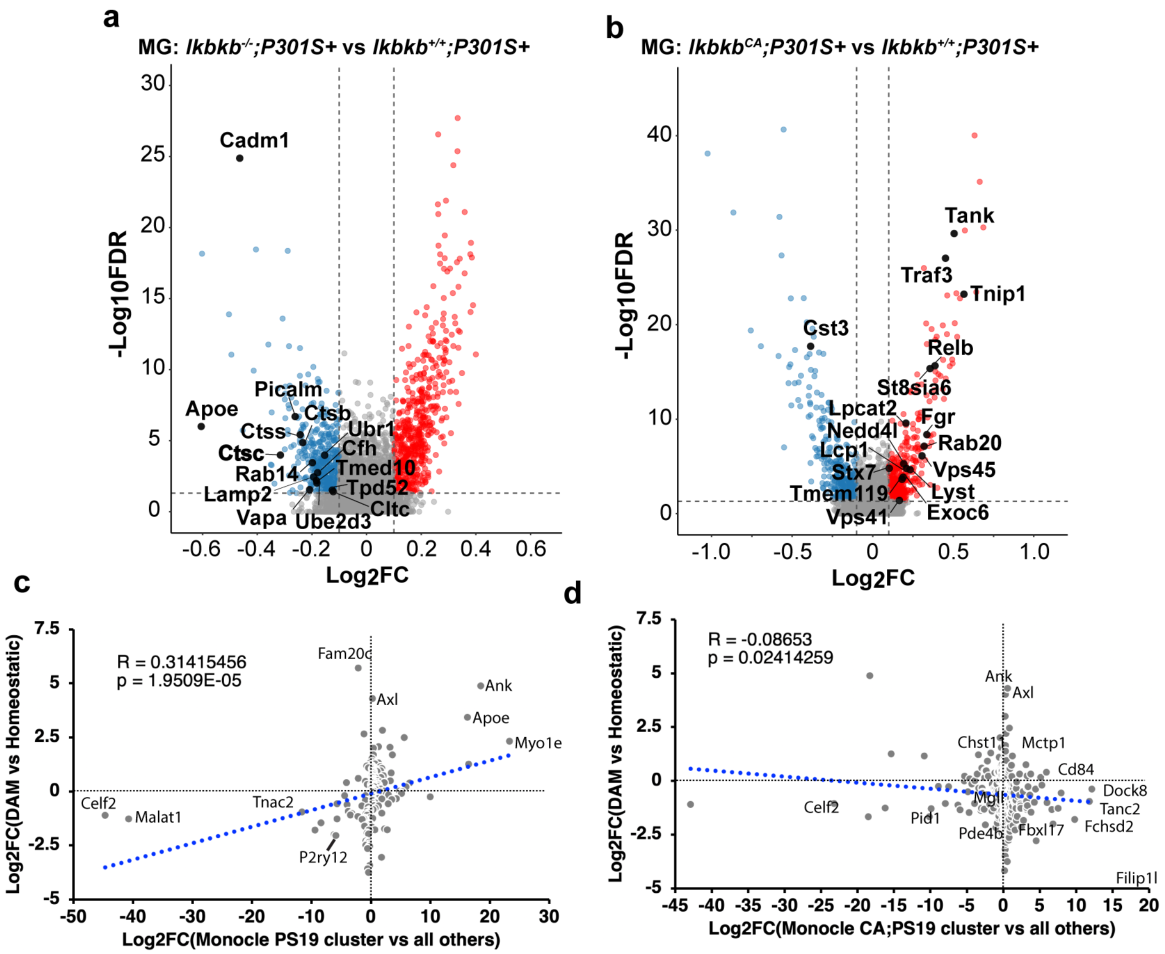

Supplementary Fig. 8 (related to Fig. 7) DEGs regulated by microglial NF-κB in PS19 mice

(a,b) Volcano plots of significant DEGs (FDR<0.05) from microglia in *Ikbkb$^{-/-}$;P301S+* mice (**a**) and *Ikbkb^{CA};P301S+* mice (**b**), in comparison to microglia from *Ikbkb$^{+/+}$;P301S+* mice. Selected DEGs related GSEA pathways are labeled.

(**c**) Correlation of genes markers of cluster 3 vs. all other clusters with DAM genes vs. homeostatic genes.

(**d**) Correlation of gene markers of cluster 4 and 5 vs. all other clusters with DAMs genes vs. homeostatic genes. P-values of (c) and (d) are calculated using Pearson correlation.

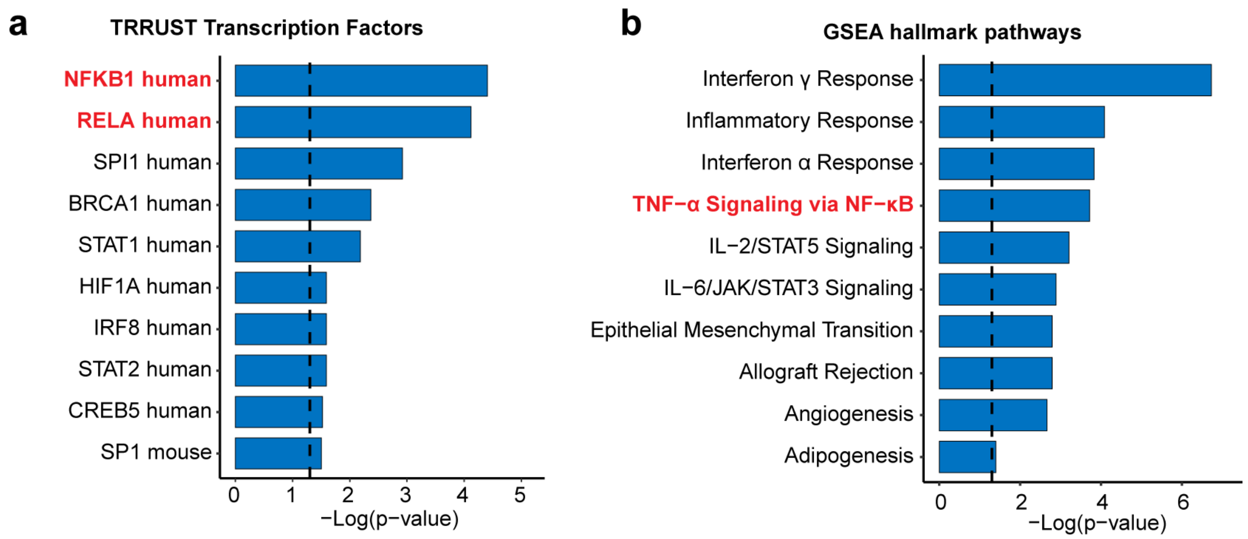

Supplementary Fig. 9 Activation of NF−κB in GSE93180: Hippocampal CD11b cells in Tau-P301S model

DEGs from GSE93180 (https://www.ncbi.nlm.nih.gov/geo/query/acc.cgi?acc=GSE93180) were analyzed by TRRUST transcription factor database (**a**) and GSEA hallmark pathways (**b**). NF−κB signaling is predicted to be activated in microglia from Tau-P301S model. P-values were calculated using right-tailed Fisher's exact test with threshold of significant enrichment as p-value≤0.05 (indicated by a dotted line of -log (p-value) = 1.3).

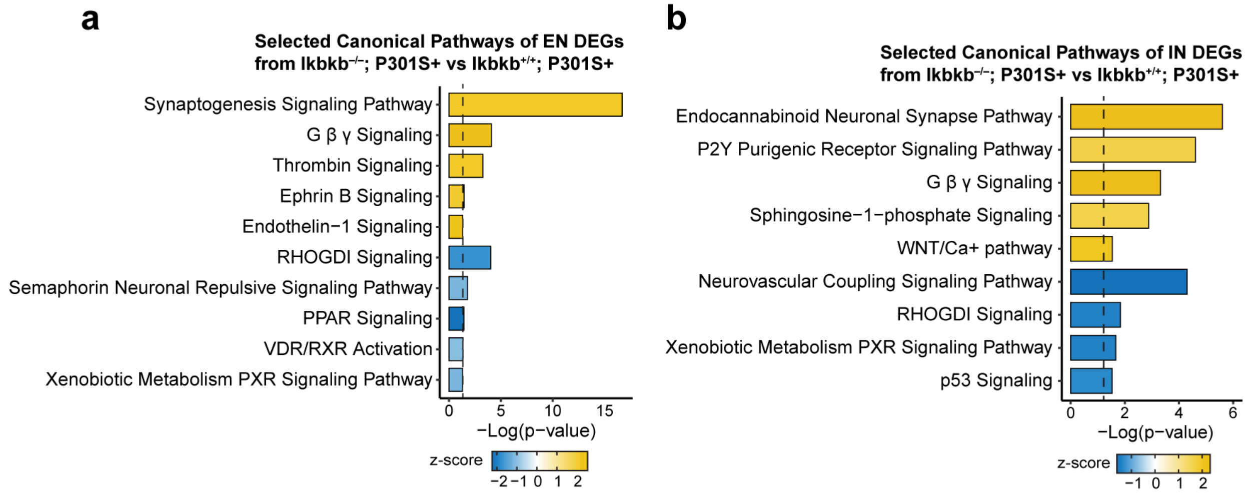

Supplementary Fig. 10 Inactivation of NF−κB alters transcriptomes in excitatory and inhibitory neurons of P301S mice

Selected IPA canonical pathways identified for DEGs of excitatory neurons (**a**) and inhibitory neurons (**b**) in *Ikbkb*$^{-/-}$*; P301S+* mice compared to *Ikbkb*$^{+/+}$*; P301S+* mice. P-values were calculated using right-tailed Fisher's exact test with threshold of significant enrichment as p-value≤0.05 (indicated by a dotted line of -log (p-value) = 1.3).
