# Supplementary material for: Microglial NF-κB drives tau spreading and toxicity in a mouse model of tauopathy
Source: Nat Commun. 2022 Apr 12;13:1969. doi: 10.1038/s41467-022-29552-6 (PMC9005658; doi:10.1038/s41467-022-29552-6)
Supplement: Supplementary file 2 — Reporting Summary [file 41467_2022_29552_MOESM2_ESM.pdf]

nature portfolio

Corresponding author(s): Li Gan

Last updated by author(s): Mar 4, 2022

Reporting Summary

Nature Portfolio wishes to improve the reproducibility of the work that we publish. This form provides structure for consistency and transparency in reporting. For further information on Nature Portfolio policies, see our Editorial Policies and the Editorial Policy Checklist.

Statistics

For all statistical analyses, confirm that the following items are present in the figure legend, table legend, main text, or Methods section.

n/a	Confirmed	
☐	☒	The exact sample size (*n*) for each experimental group/condition, given as a discrete number and unit of measurement
☐	☒	A statement on whether measurements were taken from distinct samples or whether the same sample was measured repeatedly
☐	☒	The statistical test(s) used AND whether they are one- or two-sided<br>*Only common tests should be described solely by name; describe more complex techniques in the Methods section.*
☒	☐	A description of all covariates tested
☐	☒	A description of any assumptions or corrections, such as tests of normality and adjustment for multiple comparisons
☐	☒	A full description of the statistical parameters including central tendency (e.g. means) or other basic estimates (e.g. regression coefficient) AND variation (e.g. standard deviation) or associated estimates of uncertainty (e.g. confidence intervals)
☐	☒	For null hypothesis testing, the test statistic (e.g. *F*, *t*, *r*) with confidence intervals, effect sizes, degrees of freedom and *P* value noted<br>*Give P values as exact values whenever suitable.*
☒	☐	For Bayesian analysis, information on the choice of priors and Markov chain Monte Carlo settings
☒	☐	For hierarchical and complex designs, identification of the appropriate level for tests and full reporting of outcomes
☒	☐	Estimates of effect sizes (e.g. Cohen's *d*, Pearson's *r*), indicating how they were calculated

Our web collection on statistics for biologists contains articles on many of the points above.

Software and code

Policy information about availability of computer code

Data collection	No software was used to collect data.
Data analysis	All custom codes used for snRNAseq data analysis have been archived at Zenodo (https://doi.org/10.5281/zenodo.6336233) and are directly available at https://github.com/lifan36/Wang_et_al_NatC_2021.<br>ImageJ Software (v.2.1.0) (NIH) was used for image processing and analysis. Bulk RNA-seq reads were mapped using the BlueBee genomics platform and the STAR program. The read count table was generated with the RSEM program. Differential gene expression was calculated with R package edgeR and limma. For single-nuclei RNAseq, gene counts were obtained by aligning reads to the mouse genome (mm10) with Cell Ranger software (v.3.1.0) (10x Genomics). Potential doublet cells were predicted and removed using DoubletFinder for each sample. Normalization and clustering were done with the Seurat package v3.0.1 82. Gene network and functional enrichment analysis were performed by QIAGEN's Ingenuity® Pathway Analysis (IPA®, QIAGEN Redwood City, www.qiagen.com/ingenuity) or by GSEA with molecular signatures database (MSigDB) (http://www.gsea-msigdb.org/gsea/msigdb/annotate.jsp). Statistical analyses were performed with Graphpad prism 8.0 (Graphpad, San Diego, California), STATA 12 (StataCorp) and R (R Foundation for Statistical Computing, Vienna, Austria). Data visualization were done with Graphpad and R package ggplot2.

For manuscripts utilizing custom algorithms or software that are central to the research but not yet described in published literature, software must be made available to editors and reviewers. We strongly encourage code deposition in a community repository (e.g. GitHub). See the Nature Portfolio guidelines for submitting code & software for further information.

Data

Policy information about availability of data

All manuscripts must include a data availability statement. This statement should provide the following information, where applicable:

- Accession codes, unique identifiers, or web links for publicly available datasets
- A description of any restrictions on data availability
- For clinical datasets or third party data, please ensure that the statement adheres to our policy

All data supporting this study are involved in the paper and its supplementary files. All RNA-seq data were deposited in the Gene Expression Omnibus (GEO) under the following series accession numbers: bulk-tissue RNA-seq GSE198013 [https://www.ncbi.nlm.nih.gov/geo/query/acc.cgi?acc=GSE198013]; mouse single-nuclei RNAseq, GSE198014 [https://www.ncbi.nlm.nih.gov/geo/query/acc.cgi?acc=GSE198014]. Source data are provided with this paper.

Field-specific reporting

Please select the one below that is the best fit for your research. If you are not sure, read the appropriate sections before making your selection.

☒ Life sciences ☐ Behavioural & social sciences ☐ Ecological, evolutionary & environmental sciences

For a reference copy of the document with all sections, see nature.com/documents/nr-reporting-summary-flat.pdf

Life sciences study design

All studies must disclose on these points even when the disclosure is negative.

Sample size	The sample size for in vivo and in vitro experiment was determined on the basis of previous publications and our experience. For the studies in live animals, the number of animals was determined by power analysis using information from similar studies of our publications or others' (Tau inoculation experiment was base on PMID: 23325240; microglia depletion experiment was based on our previous publication PMID: 30735499; Analysis of tau pathology and spatial learning in PS19 mice was based on PMID: 34851693, PMID: 21698260 ,PMID: 17270732). For RNAseq studies, the number of samples were determined according to our previous publications including PMID: 33054973,PMID: 30735499, PMID: 34851693,PMID: 31873194. For studies involved in brain section analysis, the number of slice was determined based on our previous studies and account for variabilities (PMID: 30735499,PMID: 34851693,PMID: 31873194). For in vitro experiments, we determined the experimental replications to account for the technical variabilities and changes with treatment based on our experience.
Data exclusions	In our analysis of Droplet-Based Single-nuclei RNA-seq, we removed genes expressed in no more than 2 cells, cells with unique gene counts over 8,000 or less than 300, and cells with high fraction of mitochondrial reads (> 5%). Potential doublet cells were predicted and removed using DoubletFinder for each sample.
Replication	All in vitro experiments were performed with a minimum of 3 biological replicates. All in vivo experiments involving behavioral tests and pathology analysis were performed with a minimum of 4 mice per genotype. The samples for snRNAseq were 2-4 mice per genotype. All attempts at replication were successful when a representative data set is provided.
Randomization	In Morris water maze test, all mice from four genotypes were randomly assigned a new number. The hidden platform training and probe trails were conducted by new numerical order. The samples in all in vivo experiments (tau inoculation and microglia depletion, snRNAseq) were randomly allocated into experimental groups in a age-matching and sex-matching manner. For in vitro experiments, control and treatment groups were attributed randomly on wells and plates and account for the location effects.
Blinding	Experimenters were blinded of genotypes when performing image analysis and quantification, including quantification of MC1 positive area, AT8+ cells, microglial number and Imaris analysis. Experimenters were blinded to group allocation during data collection and/or analysis for all of our in vivo experiments (Morris water maze test, snRNAseq, tau inoculation, microglia depletion). For in vitro experiments, experimenters labels the treatments, collected, and proceeded the samples. Thus, it is not possible for blinding. For RNAseq experiments, the samples were assigned IDs and then processed blindly. We decoded the sample identities when we integrated the datasets.

Reporting for specific materials, systems and methods

We require information from authors about some types of materials, experimental systems and methods used in many studies. Here, indicate whether each material, system or method listed is relevant to your study. If you are not sure if a list item applies to your research, read the appropriate section before selecting a response.

Materials & experimental systems

n/a	Involved in the study	
☐	☒	Antibodies
☐	☒	Eukaryotic cell lines
☒	☐	Palaeontology and archaeology
☐	☒	Animals and other organisms
☐	☒	Human research participants
☒	☐	Clinical data
☒	☐	Dual use research of concern

Methods

n/a	Involved in the study	
☒	☐	ChIP-seq
☐	☒	Flow cytometry
☒	☐	MRI-based neuroimaging

Antibodies

Antibodies used

anti-Iba1 (Fujifilm Wako, 019-19741): IHC (1:500)
anti-MC1 (a kind gift from P. Davies): IHC (1:500)
anti-phosphor-tau pS202/T205 AT8 (Thermo Fisher Scientific, # MN1020): ELISA (1ug/ml); IF (1:500)
Biotin-HT7 (Thermo Fisher Scientific, #MN1000B): ELISA(1:200)
anti-dendra2 (antibodies, Cat# ABIN361314), IF dilution: 1:1000
rat anti-lamp1(Hybridoma bank, Cat # 1d4b), IF dilution: 1:1000
Alexa Fluor 488 conjugated goat IgG against rabbit (Invitrogen: Cat # A-11034), IF dilution: 1:500
Alexa Fluor Cy5 conjugated goat IgG against rat (Invitrogen: Cat # A10525), IF dilution: 1:500
Alexa Fluor 568 conjugated goat IgG against mouse (Invitrogen# A-11004): IF (1:200)

Validation

All antibodies, except MC1, are commercial and have been chosen based on widely citation in literature. The validation/information is listed as following:
anti-Iba1 (Fujifilm Wako, 019-19741): Reliability in IHC and immunostaining is validated by many citations. (PMID: 30735499; PMID: 17270732).
anti-MC1 (a kind gift from P. Davies): Validated by Dr Davis's publication and its reliability in IHC is validated by many citations and our previous published work. PMID: 9130141,PMID: 31873194,PMID: 34851693
anti-phosphor-tau pS202/T205 AT8 (Thermo Fisher Scientific, # MN1020): this Antibody has 664 references and was verified by Cell treatment to ensure that the antibody binds to the antigen stated as described in Thermo Fisher Website (https://www.thermofisher.com/antibody/product/Phospho-Tau-Ser202-Thr205-Antibody-clone-AT8-Monoclonal/MN1020)
Biotin-HT7 (Thermo Fisher Scientific, #MN1000B): The MN1000B immunogen is purified human Tau, epitope human Tau between residue 159 and 163 (numbering according to human Tau40), corresponding to the amino acid sequence PPGQK. Its reliability in ELISA is validated by many citations. PMID: 26057852
anti-dendra2 (antibodies, Cat# ABIN361314): application in immunofluorescence independently validated for Immunocytochemistry by Neurology Lab (INSERM). PMID: 32005807.
rat anti-lamp1 (Hybridoma bank, Cat # 1d4b): application in immunofluorescence validated by original publication and its reliability is validated by many citations. PMID: 32005807

Eukaryotic cell lines

Policy information about cell lines

Cell line source(s)

Tau RD P301S FRET biosensor (ATCC#CRL-3275)

Authentication

Tau RD P301S FRET biosensor (ATCC#CRL-3275) was obtained under the material transfer agreement (MTA) for laboratory research use only. We used it to evaluate the tau seeding activity in this study, a well established assay used by many studies (PMID: 25261551).

Mycoplasma contamination

Tau RD P301S FRET biosensor (ATCC#CRL-3275) has been tested by ATCC for no myccoplasma contamination

Commonly misidentified lines
(See ICLAC register)

There was no commonly misidentified cell line was used in the study.

Animals and other organisms

Policy information about studies involving animals; ARRIVE guidelines recommended for reporting animal research

Laboratory animals

microglia depletion and K18/PL tau fibril inoculation in PS19 mice: the mice were treated at 2.5mo, tau inoculation at 3m and harvested at 4mo. Mice were housed no more than 5 per cage, given ad libitum access to food and water and were housed in a pathogen-free barrier facility with 72°F, 50% humidity, 12-hour light on/off cycle
Tau-P301S transgenic mice (P301S+,JAX:008169) or Cx3cr1CreERT2/ CreERT2 mice (JAX: 021160) were crossed with IkbkbF/F mice (MGI:2445462) or R26-StopFLikk2ca (ikbkbCAF/F) mice (JAX:008242) to obtain P301S+;IkbkbF/F, P301S+;IkbkbCAF/F, Cx3cr1CreERT2/+;IkbkbF/F, and Cx3cr1CreERT2/+;IkbkbCAF/F mice. P301S+;IkbkbF/F mice were then crossed with Cx3cr1CreERT2/+;IkbkbF/F mice to obtain P301S+;Cx3cr1CreERT2/+;IkbkbF/F mice and littermates controls including IkbkbF/F, Cx3cr1CreERT2/+;IkbkbF/F, and P301S+;IkbkbF/F mice. Similarly, P301S+;IkbkbCAF/F mice were then crossed with Cx3cr1CreERT2/+; IkbkbCAF/F mice to obtain P301S+;Cx3cr1CreERT2/+;IkbkbCAF/F mice and littermates controls including IkbkbCAF/F, Cx3cr1CreERT2/+; IkbkbCAF/F, and P301S+;

	IkbkbCAF/F mice.<br>1) Figure 4: Tamoxifen induction started from 2.5mo, tau inoculation at 3mo and harvested at 4mo. 2) Figure 5 and Figure 6: mice were tested for spatial memory in 8-9mo and harvested at 9-10mo. 3) Figure 7: SnRNAseq, 9-10mo.<br>Mice of both sexes were used for all experiment except for RNA-seq, which used only male mice.
Wild animals	no wild animals were used in the study.
Field-collected samples	no field collected samples were used in the study.
Ethics oversight	All animal work was performed in accordance with NIH guidelines and protocols approved by University of California, San Francisco, Institutional Animal Care and Use Committee.

Note that full information on the approval of the study protocol must also be provided in the manuscript.

Human research participants

Policy information about studies involving human research participants

Population characteristics	*Describe the covariate-relevant population characteristics of the human research participants (e.g. age, gender, genotypic information, past and current diagnosis and treatment categories). If you filled out the behavioural & social sciences study design questions and have nothing to add here, write "See above."*
Recruitment	*Describe how participants were recruited. Outline any potential self-selection bias or other biases that may be present and how these are likely to impact results.*
Ethics oversight	*Identify the organization(s) that approved the study protocol.*

Note that full information on the approval of the study protocol must also be provided in the manuscript.

Flow Cytometry

Plots

Confirm that:

- [x] The axis labels state the marker and fluorochrome used (e.g. CD4-FITC).
- [x] The axis scales are clearly visible. Include numbers along axes only for bottom left plot of group (a 'group' is an analysis of identical markers).
- [x] All plots are contour plots with outliers or pseudocolor plots.
- [x] A numerical value for number of cells or percentage (with statistics) is provided.

Methodology

Sample preparation	In brief, mouse brain tissue was placed in 1500 µl of Sigma nuclei PURE lysis buffer (Sigma, NUC201-1KT) and homogenized with a Dounce tissue grinder (Sigma, D8938-1SET) with 15 strokes with pestle A and 15 strokes with pestle B. The homogenized tissue was filtered through a 35-µm cell strainer and were centrifuged at 600 g for 5 min at 4°C and washed three times with 1 ml of PBS containing 1% BSA, 20 mM DTT and 0.2 U µl–1 recombinant RNase inhibitor. Then the nuclei were centrifuged at 600 g for 5 min at 4°C and re-suspended in 500 µl of PBS containing 0.04% BSA and 1x DAPI.
Instrument	Sony Corporation, LE-MA900FP
Software	Sony Corporation, Cell Sorter Software v3.2
Cell population abundance	10.45% of total events were DAPI+ nuclei using DAPI-A vs DAPI-H.
Gating strategy	Most events were included in the FSC/SSC gate. Singlet, DAPI-stained nuclei were sorted using DAPI-A vs DAPI-H.

- [x] Tick this box to confirm that a figure exemplifying the gating strategy is provided in the Supplementary Information.
