# Supplementary material for: Microglial NF-κB drives tau spreading and toxicity in a mouse model of tauopathy
Source: Nat Commun. 2022 Apr 12;13:1969. doi: 10.1038/s41467-022-29552-6 (PMC9005658; doi:10.1038/s41467-022-29552-6)
Supplement: Supplementary file 4 — Description of Additional Supplementary Files [file 41467_2022_29552_MOESM4_ESM.pdf]

Title: Supplementary Data 1.
Description: Endotoxin levels of tau monomer and fibrils.

Title: Supplementary Data 2.
Description: The differentially expressed genes (FDR<0.05) in primary microglia treated with full length tau fibrils (n=4) in comparison to vehicle control (n=4).

Title: Supplementary Data 3.
Description: DEGs list (highlighted in blue) for each IPA canonical pathway shown in Figure 1b.

Title: Supplementary Data 4.
Description: The differentially expressed genes (FDR<0.05) in isolated microglia from 11-month-old PS19 mice (n=4) in comparison to non-transgenic control (n=4).

Title: Supplementary Data 5.
Description: The differentially expressed genes (FDR<0.05) both changed in tau fibrils stimulated primary microglia and microglia isolated from 11-month-old PS19 mice in comparison to their corresponding controls.

Title: Supplementary Data 6.
Description: DEGs list (highlighted in blue) for each IPA canonical pathway shown in Figure 1f.

Title: Supplementary Data 7.
Description: The differentially expressed genes (FDR<0.05) both changed in ikbkbCA microglia and tau fibrils stimulated primary microglia in comparison to their corresponding controls.

Title: Supplementary Data 8.
Description: DEGs list (highlighted in blue) for each IPA canonical pathway shown in Figure 2e.

Title: Supplementary Data 9_1.
Description: The differentially expressed genes (FDR<0.05) uniquely changed in ikbkb-/- primary microglia (n=4) in comparison to ikbkb+/+ microglia (n=4).

Title: Supplementary Data 9_2.
Description: The differentially expressed genes (FDR<0.05) uniquely changed in ikbkbCA primary microglia (n=5) in comparison to ikbkbWT microglia (n=5).

Title: Supplementary Data 10-1.
Description: DEGs list (highlighted in blue) for each IPA canonical pathway shown in Supplementary Figure 2a.

Title: Supplementary Data 10-2.
Description: DEGs list (highlighted in blue) for each IPA canonical pathway shown in Supplementary Figure 2b.

Title: Supplementary Data 10-3.
Description: DEGs list (highlighted in blue) for each IPA canonical pathway shown in Supplementary Figure 2c.

Title: Supplementary Data 11.
Description: The differentially expressed genes (FDR<0.05) both changed in ikbkbCA and ikbkb-/-primary microglia in comparison to their corresponding controls.

Title: Supplementary Data 12.
Description: The upregulated differentially expressed genes (FDR<0.05) in cortical tissues of IkbkbCA mice (n=4) in comparison to IkbkbWT mice (n=4).

Title: Supplementary Data 13.
Description: The differentially expressed genes (FDR<0.05) in cortical tissues of ikbkb+/+;P301S+ mice (n=4) but not in ikbkb-/-;P301S+ mice (n=4), in comparison to ikbkb+/+ mice (n=4).

Title: Supplementary Data 14.
Description: DEGs list (highlighted in blue) for each IPA canonical pathway shown in Figure 6f.

Title: Supplementary Data 15.
Description: The signature differentially expressed genes (p_val_adj<0.05) from 5 monocle microglia subclusters shown in Figure 7C. avg_logFC: log fold-chage of the average expression between the two groups; p_val_adj: Adjusted p-value, based on bonferroni correction using all genes in the dataset.

Title: Supplementary Data 16-1.
Description: The snRNA-seq differentially expressed genes (FDR<0.05, Log2FC>0.1 or <-0.1) in microglia from ikbkb-/- P301S+ mouse coritical tissues (n=2) in comparison to ikbkb+/+ P301S+ mice (n=3).

Title: Supplementary Data 16-2.
Description: The snRNA-seq differentially expressed genes (FDR<0.05, Log2FC>0.1 or <-0.1) in microglia from ikbkbCA;P301S+ mouse coritical tissues (n=2) in comparison to ikbkb+/+;P301S+ mice (n=3).

Title: Supplementary Data 17.
Description: The differentially expressed genes (p_val_adj<0.05) from monocle microglia subcluster 3 vs subcluster 1 and from subcluster 4 and 5 vs subcluster 1 shown in figure 7g. The overlap 122 upregulated DEGs is shown in tab'Overlap DEGs Up' avg_logFC: log fold-chage of the average expression between the two groups; p_val_adj: Adjusted p-value, based on bonferroni correction using all genes in the dataset.

Title: Supplementary Data 18.
Description: DEGs in GSE93180: Hippocampal CD11b cells in Tau-P301S model. log2(fold

change): log fold-change of the average expression between the two groups using the Wald test. Adjusted P-value: adjusted p-value for multiple testing using the Benjamini and Hochberg method.
